# Three-port approach vs conventional laparoscopic radical cystectomy with orthotopic neobladder: a single-center retrospective study

**DOI:** 10.1186/s12957-023-03031-8

**Published:** 2023-05-25

**Authors:** Shuxin Dai, Chang Liu, Zhiwei Jiang, Xiangyu Teng, Songbai Yan, Dian Xia, Zhouting Tuo, Xin Wang, Qi Wang, Liangkuan Bi

**Affiliations:** 1 Department of Urology, The Second Affiliated Hospital of Anhui Medical University, NO. 678 Furong Road, Hefei, 230032 China; 2 Peking University Shenzhen Hospital, Shenzhen, China

**Keywords:** Three-port, Five-port, Laparoscopic radical cystectomy, Surgical treatment, Bladder cancer

## Abstract

**Background:**

This study aimed to compare the clinical outcomes of patients who underwent three-port laparoscopic radical cystectomy (LRC) with orthotopic neobladder (ONB) and traditional five-port method.

**Methods:**

From January 2017 to November 2020, 100 patients underwent LRC + ONB at a third-level grade A hospital.

**Results:**

Our study included 55 patients who underwent three-port LRC and 45 patients who underwent the five-port method. There were no significant differences in perioperative data such as operation time (253.00 ± 43.89 vs. 259.07 ± 52.31 min, *P* = 0.530), estimated blood loss (EBL)(97.64 ± 59.44 vs. 106.67 ± 55.35 min, *P* = 0.438), day to flatus (2.25 ± 1.49 vs. 2.76 ± 1.77 days, *P* = 0.128), day to regular diet (7.07 ± 2.99 vs. 7.96 ± 3.32 days, *P* = 0.165), day to pelvic drain removal (9.58 ± 3.25 vs. 10.53 ± 3.80 days, *P* = 0.180), and hospital stay after operation (11.62 ± 3.72 vs. 11.84 ± 4.37 days, *P* = 0.780) between the two groups. The only significant difference was in the treatment cost (*P* = 0.035). Similarly, postoperative complications, quality of life, and tumor outcomes were not significantly different between the two groups (*P* > 0.05).

**Conclusions:**

The three-port method is safe and feasible for patients suitable for traditional five-port LRC with an orthotopic neobladder.

## Background

As a common malignant tumor in urology, the detection rate of bladder cancer has increased significantly with the development of the social economy and medicine. Musculo-invasive bladder cancer is difficult to treat because of its highly malignant biological behavior and tendency for distant metastasis. Radical cystectomy (RC) remains the gold standard and preferred method for treating muscle-invasive bladder cancer (MIBC) and high-risk non-muscle-invasive bladder cancer (NMIBC) with multiple recurrence [1, 2]. The probability of complications in the perioperative period and within 3 months after RC can reach 20–60.4% [3], making it one of the most difficult operations in urology. Radical surgery for bladder cancer has a long history. RC was first reported by Marshall et al. [4] in 1949 and has since been developed. Through the development of open radical cystectomy (ORC), LRC, and robot-assisted radical cystectomy (RARC) [5, 6], treatment has been standardized. Among them, RARC remains popular due to fewer complications, better postoperative recovery, and comparable treatment effects with ORC [6–8]. However, for many patients in developing countries and those with low economic levels, high operation costs makes RARC inaccessible [9, 10]. In such cases, LRC may be a more economical and reasonable choice. Since Parra et al. [11] first reported their attempted LRC in 1992, traditional LRC with five ports have been widely used [12]. With the development of medicine, the improvement of surgeons' skills, and people's demand for aesthetics, laparoscopic surgery is developing towards reducing the number of cannulas [13]. This is also the general trend with minimally invasive surgery. LRC should also aim to reduce the number of casings because more these may lead to more complications and financial costs. Three-port LRC is simplified and optimized on the basis of a five-port LRC, and this innovative surgical method was adopted and improved by Professor Bi from our research group [14, 15]. In this study, we analyzed preoperative, intraoperative, and postoperative data from three-port LRC and the conventional five-port method to study their clinical significance and practical value.

## Methods

### Patients

After obtaining ethical approval from the Ethics Committee of Anhui Medical University, we selected 100 patients who underwent LRC + ONB in our hospital from January 2017 to November 2020 for a retrospective study design and analysis. We introduced both methods to each patient and did not intentionally recommend either the three-port or the five-port procedure. Because some patients were reluctant to undergo the innovative three-port procedure and others were reluctant to undergo more abdominal incisions, we performed different numbers of cannulas in different patients. There were 55 patients in the three-port group and 45 in the five-port group. These data were obtained from the patient information database at our hospital. The inclusion criteria of this study included the following: (1) patients had a preoperative pathological diagnosis of MIBC or high-grade NMIBC; (2) all operations were performed by the same surgeon (Professor Bi) in the Second Affiliated Hospital of Anhui Medical University; (3) only three trocars were used from the beginning to the end of the procedure; (4) patients had good preoperative urinary control, no history of intestinal disease or intestinal resection, and no distant metastasis. and (5) the medical records were complete [14–16]. The inclusion criterion was muscle-invasive or recurrent high-risk bladder tumors that did not respond to intravesical immunotherapy. Patients with distant metastasis, severe cardiopulmonary dysfunction, positive urethral margins, active enteritis, and renal dysfunction were excluded [14–16]. All patients underwent B-ultrasonography, CT, MRI, cystoscopy, and other detailed examinations before the operation, and all of them provided written informed consent. All operations were performed by Dr. Bi of our research group. The preoperative demographic characteristics are shown in Table 1.

**Table 1:** Preoperative clinical characteristics of 100 bladder cancer patients

**Characteristic**	**Overall (*n* = 100)**	**Three-**port** group (*n* = 55)**	**Five-**port** group (*n* = 45)**	***P* value**
Age, [years, mean ± SD (range)]	66.89 ± 12.16 (31–91)	67.04 ± 12.19 (31–91)	66.71 ± 12.26 (40–90)	0.895
Gender, [n (%)]				0.451
Male	86 (86.00)	46 (83.64)	40 (88.89)	
Female	14 (14.00)	9 (16.36)	5 (11.11)	
Year of surgery interval, [n (%)]				0.884
2017–2018	37 (37.00)	20 (36.36)	17 (37.78)	
2019–2020	63 (63.00)	35 (63.64)	28 (62.22)	
BMI, [kg/m2, mean ± SD (range)]	23.12 ± 3.40 (15.06–31.18)	23.04 ± 3.27 (15.06–29.07)	23.20 ± 3.59 (15.39–31.18)	0.815
Smoking status, [n (%)]				0.412
Yes	40 (40.00)	20 (36.36)	20 (44.44)	
No	60 (60.00)	35 (63.64)	25 (55.56)	
Diabetes mellitus, [n (%)]				0.654
Yes	42 (42.00)	22 (40.00)	20 (44.44)	
No	58 (58.00)	33 (60.00)	25 (55.56)	
Hypertension, [n (%)]				0.317
Yes	41 (41.00)	25 (45.45)	16 (35.56)	
No	59 (59.00)	30 (54.55)	29 (64.44)	
Hematuria, [n (%)]				0.867
Yes	77 (77.00)	42 (76.36)	35 (77.78)	
No	23 (23.00)	13 (23.64)	10 (22.22)	
Previous abdominal surgery, [n (%)]				0.700
Yes	27 (27.00)	14 (25.45)	13 (28.89)	
No	73 (73.00)	41 (74.55)	32 (71.11)	
Previous TURBT, [n (%)]				0.821
Yes	39 (39.00)	22 (40.00)	17 (37.78)	
No	61 (61.00)	33 (60.00)	28 (62.22)	
Previous chemotherapy, [n (%)]				0.551
Yes	26 (26.00)	13 (23.64)	13 (28.89)	
No	74 (74.00)	42 (76.36)	32 (71.11)	
Preoperative hydronephrosis, [n (%)]				0.818
Yes	19 (19.00)	10 (18.18)	9 (20.00)	
No	81 (81.00)	45 (81.82)	36 (80.00)	
Preoperative creatinine, [umol/L, median (range)]	86.50 (35.00–161.00)	87 (35.00–161.00)	85 (37.00–158.00)	0.717
ASA score, [n (%)]				0.273
I	15 (15.00)	11 (20.00)	4 (8.89)	
II	62 (62.00)	33 (60.00)	29 (64.44)	
III	23 (23.00)	11 (20.00)	12 (26.67)	
Clinical tumor stage, [n (%)]				0.959
≤ T1	9 (9.00)	5 (9.09)	4 (8.89)	
T2	51 (51.00)	29 (52.73)	22 (48.89)	
T3	35 (35.00)	18 (32.73)	17 (37.78)	
T4	5 (5.00)	3 (5.45)	2 (4.44)	

### Surgical technique

**Fig. 1:**
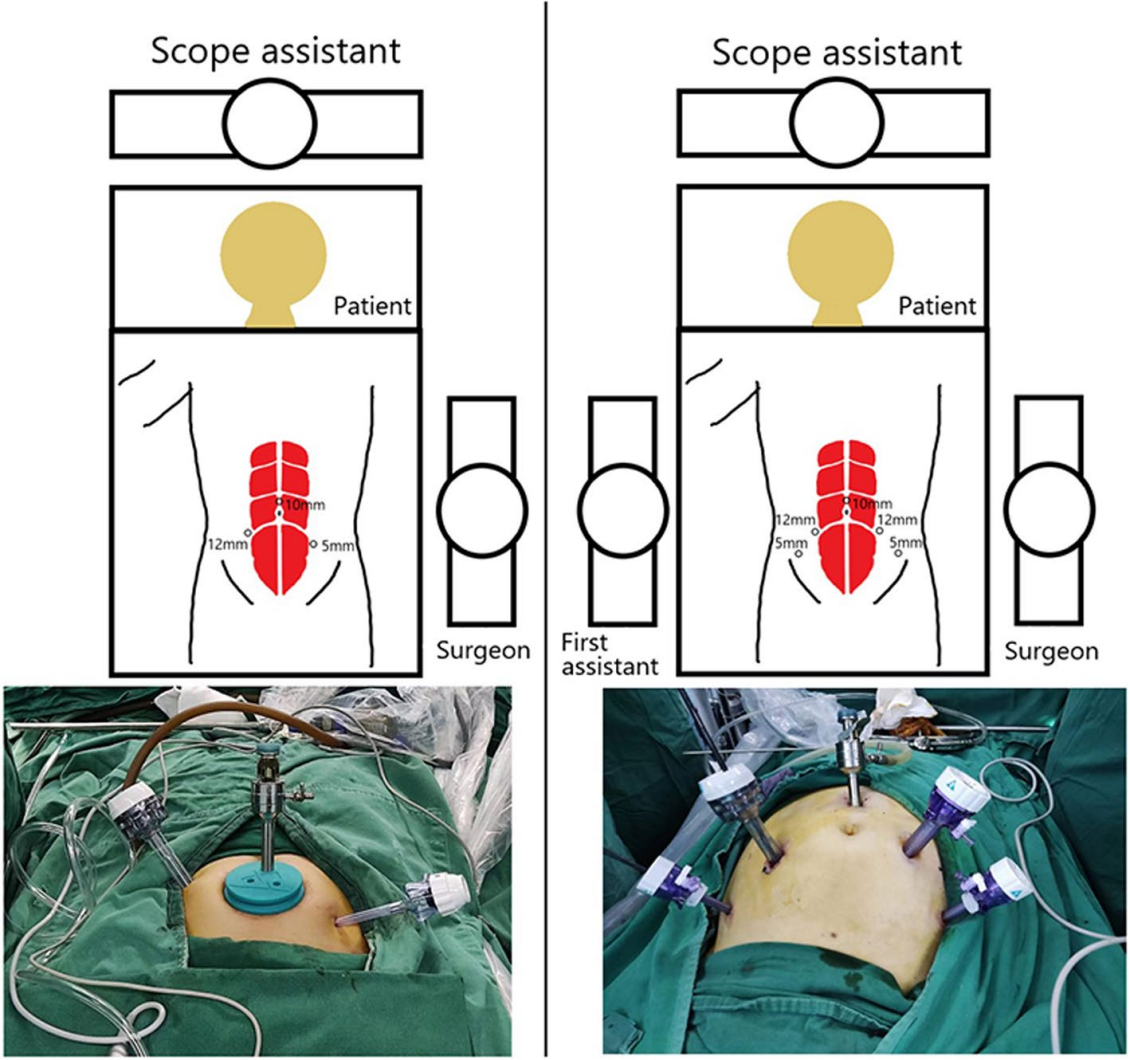
Two surgical methods of port placement. The left side shows the casing position for the three-port method, and the right side shows the casing position for the five-port method

All operations were performed in strict accordance with standardized procedures. The five-port LRC we used was a traditional operation, whereas the three-port method was detailed in our previous study [14]. The detailed operation video can be watched on https://pan.baidu.com/s/1e82AB4OVtS5roSbiRTmeIg (extraction code: eryi). Preoperative preparation of the patient included a 12-h fast from water, an enema, and oral antibiotics for one day. As shown in Fig. 1, our three-port LRC requires only one surgeon standing on the patient's left side and a laparoscopic assistant standing on the patient’s head. We used the STORZ brand laparoscopic platform for all operations. The observation hole for laparoscopy was located 2 cm above the navel. The 12 mm main surgical hole was located on the right rectus abdominis muscle, and 4 cm below the navel. The auxiliary 5 mm surgical hole was located on the left side of the patient above the rectus abdominis muscle and 5–7 cm below the umbilicus. After successful anesthesia, the cannula was placed in two ways, as shown in Fig. 1. The bladder and prostate were removed, and pelvic lymph nodes were dissected. Finally, Studer ONB and urethral reconstruction were performed. All patients were admitted to the ICU to receive good postoperative care, and they usually returned to the general ward one day later. The patient received bladder irrigation on the first postoperative day, and 200 ml of normal saline was used to irrigate the bladder in the morning and evening. We instructed the patient to ambulate early and resume eating according to bowel conditions. About 30 days after surgery, the double J tubes were removed under cystoscope [14].

### Follow-up and data collection

All patients who underwent surgery were followed-up by telephone or in the clinic, and their data were obtained. The follow-up times were 1, 3, 6, 9, and 12 months after surgery, and every six months thereafter. Routine blood urine, renal function tests, CT scan, cytological examination, as well as urinary system B ultrasound, and other tests were performed at each follow-up. Postoperative Urination, quality of life, and tumor outcomes were regularly followed over a 2-year period.

We collected relevant clinical data of 100 patients, including preoperative data such as age, sex, and body mass index (BMI); perioperative data such as operation method, operation time, and intraoperative blood loss; and postoperative data such as postoperative complications, urinary incontinence, and tumor outcome.

### Statistical analysis

SPSS (version 26.0; IBM, Armonk, NY, USA) was used for data analysis. Categorical variables were expressed as the number of cases and percentages. Differences between groups were compared using the chi-square test or Fisher’s exact test. Continuous variables were expressed as mean ± standard deviation, and differences were compared using Student's t-test. A two-sided *P*-value of less than 0.05 was considered statistically significant.

## Results

### Patient characteristics

Of the 100 patients with MIBC and high-risk NMIBC in this study, 55 underwent three-port LRC while 45 underwent five-port LRC. Operating times ranged from 2017 to 2020, with most surgeries performed in 2019–2020. We collected a series of preoperative clinical data for these patients, as shown in Table 1, including age, sex, operation year, BMI, smoking history, diabetes mellitus, hypertension, hematuria, abdominal surgery history, TURBT history, chemotherapy history, preoperative hydronephrosis, preoperative creatinine level, ASA score, and clinical tumor stage. There was no significant difference in preoperative clinical data between the two groups (*P* > 0.05).

### Operative and pathological outcomes

**Fig. 2:**
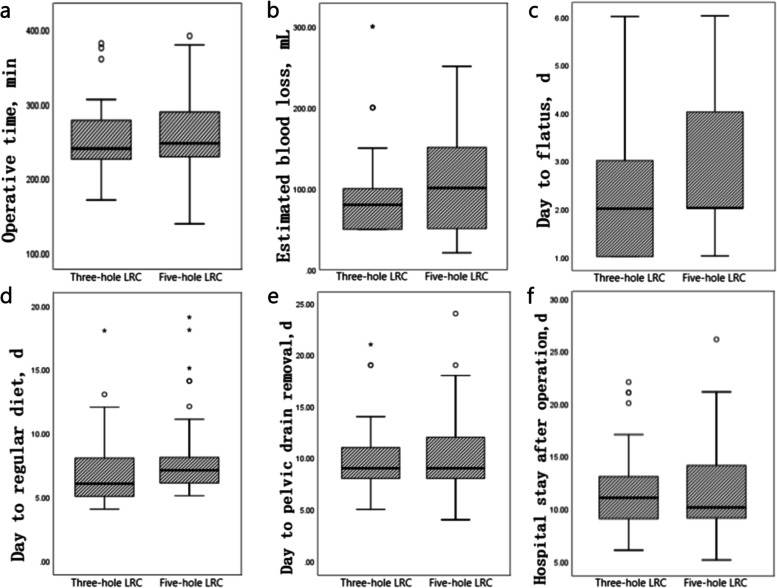
Comparison of some perioperative clinical data between the two groups. **a** Contrast of OT. **b** Contrast of EBL. **c** Contrast of day to flatus. **d** Contrast of day to regular diet. **e** Contrast of day to pelvic drain removal. **f** Contrast of hospital stay after operation

Perioperative data of 100 patients are presented in Table 2. We counted the operation time (OT), estimated blood loss (EBL), transfusion rate, days to flatus, days to regular diet, days to pelvic drain removal, hospital stay after surgery, pathology type, pathologic T stage, pathologic N stage, and mean treatment cost. The operative time (253.00 ± 43.89 vs. 259.07 ± 52.31 min, *P* = 0.530) (Fig. 2a), EBL (97.64 ± 59.44 vs. 106.67 ± 55.35 min, *P* = 0.438) (Fig. 2b), day to flatus (2.25 ± 1.49 vs. 2.76 ± 1.77 days, *P* = 0.128) (Fig. 2c), day to regular diet (7.07 ± 2.99 vs. 7.96 ± 3.32 days, *P* = 0.165) (Fig. 2d), day to pelvic drain removal (9.58 ± 3.25 vs. 10.53 ± 3.80 days, *P* = 0.180) (Fig. 2e), and hospital stay after operation (11.62 ± 3.72 vs. 11.84 ± 4.37 days, *P* = 0.780) (Fig. 2f) were not significantly different between the two groups. None of the surgical margins showed any positive results. The pathological type of the tumor was mainly transitional cell carcinoma(98.00%), and only two cases were squamous cell carcinoma (2.00%). Additionally, there was no significant difference in tumor stage between them (*P* > 0.05). These data showed that there was no significant difference in perioperative clinical data and postoperative recovery between the two groups (all *P* > 0.05). Moreover, we calculated the median treatment cost of the two groups of patients and found these to be significant difference (*P* = 0.035).

**Table 2:** Perioperative outcomes of 100 bladder cancer patients

**Outcomes**	**Overall (*n* = 100)**	**Three-**port** group (*n* = 55)**	**Five-**port** group (*n* = 45)**	***P* value**
Operative time, [min, mean ± SD (range)]	255.73 ± 47.71 (138–390)	253.00 ± 43.89 (171–381)	259.07 ± 52.31 (138–390)	0.530
Estimated blood loss (EBL), [mL, mean ± SD (range)]	101.70 ± 57.53 (20–300)	97.64 ± 59.44 (50–300)	106.67 ± 55.35 (20–250)	0.438
Transfusion rate, [n (%)]	10 (10.00)	5 (9.09)	5 (11.11)	0.738
Day to flatus, [d, mean ± SD (range)]	2.48 ± 1.64 (1–6)	2.25 ± 1.49 (1–6)	2.76 ± 1.77 (1–6)	0.128
Day to regular diet, [d, mean ± SD (range)]	7.47 ± 3.15 (4–20)	7.07 ± 2.99 (4–20)	7.96 ± 3.32 (5–19)	0.165
Day to pelvic drain removal, [d, mean ± SD (range)]	10.01 ± 3.52 (4–24)	9.58 ± 3.25 (5–21)	10.53 ± 3.80 (4–24)	0.180
Hospital stay after operation, [d, mean ± SD (range)]	11.72 ± 4.01 (5–26)	11.62 ± 3.72 (6–22)	11.84 ± 4.37 (5–26)	0.780
Pathology type, [n (%)]				0.886
Transitional cell carcinoma	98 (98.00)	54 (98.18)	44 (97.78)	
Squamous cell carcinoma	2 (2.00)	1 (1.82)	1 (2.22)	
Lymph node yield, [n, mean ± SD]	18.13 ± 2.63	17.78 ± 3.03	18.56 ± 1.98	0.144
Lymph node positive, [n (%)]	9 (9.00)	5 (9.09)	4 (8.89)	0.626
Pathologic T stage, [n (%)]				0.872
≤ T1	17 (17.00)	8 (14.55)	9 (20.00)	
T2	37 (37.00)	20 (36.36)	17 (37.78)	
T3	41 (41.00)	24 (43.64)	17 (37.78)	
T4	5 (5.00)	3 (5.45)	2 (4.44)	
Pathologic N stage, [n (%)]				0.577
N0	91 (91.00)	50 (90.91)	41 (91.11)	
N1	4 (4.00)	3 (5.45)	1 (2.22)	
N2	5 (5.00)	2 (3.64)	3 (6.67)	
Mean treatment cost [$, mean (range)]^a^	10,894.85 (6455.00–28,730.6)	10,087.56 (6455.00–26,921.35)	11,755.62 (7921.75–28,730.61)	0.035

^a^We converted the treatment cost based on 2022 currency exchange rates

### Postoperative outcomes

Postoperative outcomes are shown in Table 3, and postoperative complications according to the Clavien-Dindo classification are shown in Table 4. The median follow-up time for all patients in our study group was 24 months, including 24 months (6–36 months) in the three-port group, and 24 months (6–32 months) in the five-port group. Incision problems (9%), infectious fever (9%), and gastrointestinal complications (8%) were common early complications. Meanwhile, hydronephrosis (9%) and urethral stricture (8%) were the most common late complications. There were no significant differences calculated for either the Clavien-Dindo classification (Table 4) or the more specific classification of various complications (Table 3) (*P* > 0.05).

**Table 3:** Postoperative outcomes of 100 bladder cancer patients

**Variables**^a^	**Overall (*n* = 100)**	**Three-**port** group (*n* = 55)**	**Five-**port** group (*n* = 45)**	***P* value**
hydronephrosis, [n (%)]	9 (9.00)	4 (7.27)	6 (13.33)	0.315
Urinary tract stricture, [n (%)]	8 (8.00)	4 (7.27)	5 (11.11)	0.505
Bowel obstruction, [n (%)]	8 (8.00)	3 (5.45)	5 (11.11)	0.300
Febrile urinary tract infection, [n (%)]	9 (9.00)	3 (5.45)	6 (13.33)	0.171
Wound dehiscence, [n (%)]	4 (4.00)	2 (3.64)	2 (4.44)	0.837
Wound infection, [n (%)]	5 (5.00)	2 (3.64)	3 (6.67)	0.489
Intestinal or urinary fistula, [n (%)]	5 (5.00)	3 (5.45)	2 (4.44)	0.818
Diarrhea, [n (%)]	6 (6.00)	3 (5.45)	3 (6.67)	0.800
Incisional hernia, [n (%)]	4 (4.00)	3 (5.45)	1 (2.22)	0.412
Vein thrombosis, [n (%)]	2 (2.00)	1 (1.82)	1 (2.22)	0.886
Sepsis, [n (%)]	2 (2.00)	1 (1.82)	1 (2.22)	0.886

^a^The same patient may have multiple complications

**Table 4:** Postoperative complications according to Clavien-Dindo classification

**Complications**^a^	**Overall (*n* = 100)**	**Three-**port** group (*n* = 55)**	**Five-**port** group (*n* = 45)**	***P* value**
Complication occurred patients, [n (%)]	53 (53.00)	28 (50.91)	25 (55.56)	0.643
Early complication (Clavien-Dindo classification) [n, % of patients undergoing early complications]	49 (49.00)	25 (45.45)	24 (53.33)	0.605
1	21 (21.00)	13 (23.64)	8 (17.78)	
2	19 (19.00)	8 (14.55)	11 (24.44)	
3a	5 (5.00)	2 (3.64)	3 (6.67)	
3b	4 (4.00)	2 (3.64)	2 (4.44)	
Late complication (Clavien-Dindo classification) [n, % of patients undergoing late complications]	37 (37.00)	19 (34.55)	18 (40.00)	0.915
1	7 (7.00)	4 (7.27)	3 (6.67)	
2	12 (12.00)	6 (10.91)	6 (13.33)	
3a	15 (15.00)	7 (12.73)	8 (17.78)	
3b	3 (3.00)	2 (3.64)	1 (2.22)	

^a^The same patient may have both early and late complications

### Oncologic and functional outcomes

As shown in Table 5, postoperative urinary incontinence and tumor outcomes were analyzed. Day-and night-controlled urination were defined as the use of a pad no more than once a day (0–1 pad/day). In contrast, more than one (> 1pad/day) is considered uncontrolled urination. Twelve months postoperatively, the rate of daytime incontinence was 85.45% in the three-port group and 86.67% in the five-port group (*P* = 0.862). The rates of nighttime incontinence were 65.45% and 68.89% in the two groups, respectively (*P* = 0.716). After two years of follow-up, there were two deaths from recurrent bladder cancer in each group, and three and four deaths from other causes, respectively. In our 2-year study, no significant differences were observed in cancer-specific mortality, non-cancer-specific mortality, and neobladder capacity (*P* > 0.05).

**Table 5:** Oncological and functional outcomes of 100 bladder cancer patients

**Variables**	**Overall (*n* = 100)**	**Three-**port** group (*n* = 55)**	**Five-**port** group (*n* = 45)**	***P* value**
Cancer-specific mortality for 2 years, [n (%)]	4 (4.00)	2 (3.64)	2 (4.44)	0.837
Noncancer-specific mortality for 2 years, [n (%)]	7 (7.00)	3 (5.45)	4 (8.89)	0.503
Daytime incontinence at 12 months, [n (%)]				0.862
0–1 pad/day	86 (86.00)	47 (85.45)	39 (86.67)	
> 1 pad/day	14 (14.00)	8 (14.55)	6 (13.33)	
Nighttime incontinence at 12 months, [n (%)]				0.716
0–1 pad/day	67 (67.00)	36 (65.45)	31 (68.89)	
> 1 pad/day	33 (33.00)	19 (34.55)	14 (31.11)	
Neobladder capacity at 12 months, [mean ± SD (range), mL]	421.10 ± 46.56 (360–500)	425.45 ± 51.24 (360–500)	415.78 ± 40.03 (360–480)	0.303

### Health-related quality of life results

**Fig. 3:**
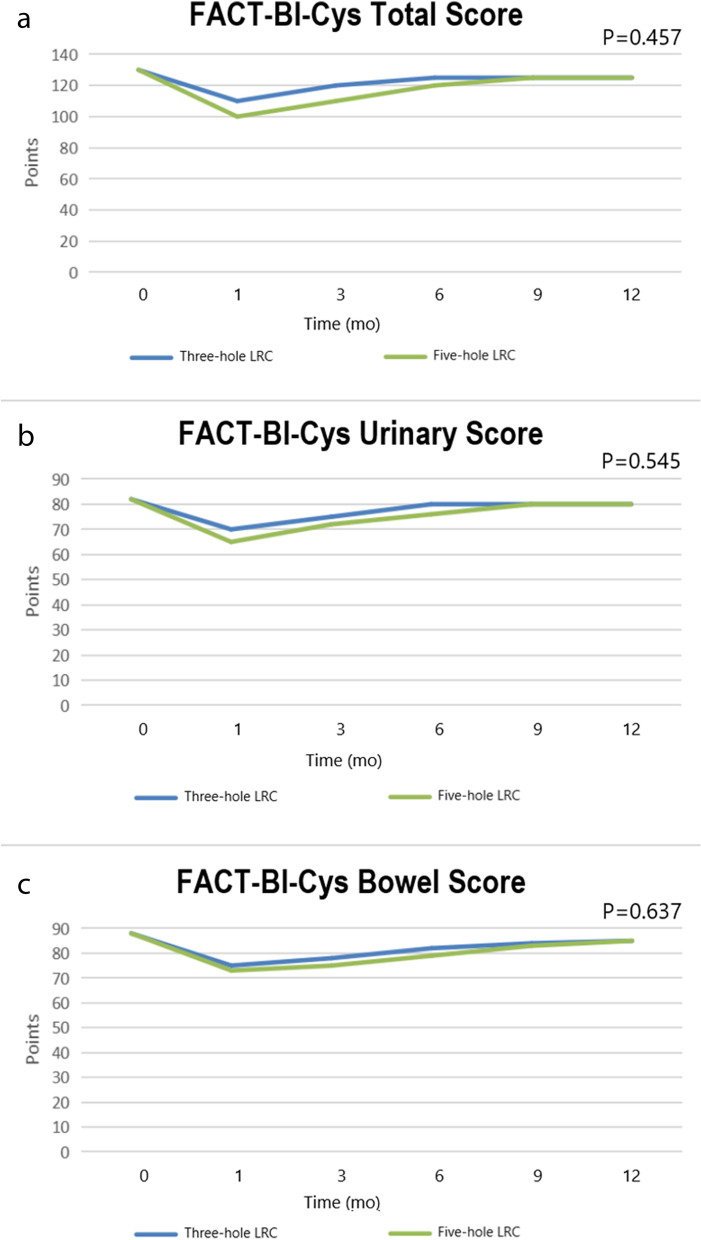
IPW-adjusted mean HRQOL scores before cystectomy and at 1, 3, 6, 9, and 12 months. **a** FACT-Bl-Cys (Functional Assessment of Cancer Therapy-Bladder Cystectomy) Total Score. **b** BCI (Bladder Cancer Index) urinary domain scores. **c** BCI bowel domain scores

We evaluated the quality of life of these patients before surgery and at 1, 3, 6, 9, and 12 months after surgery by telephone follow-up, outpatient follow-up, questionnaires, and other means. After statistical analysis, Fig. 3 was drawn. According to the images drawn by Bladder Cancer Index (BCI), Functional Assessment of Cancer Therapy-Bladder Cystectomy (FACT-Bl-Cys) and time, the broken line corresponding to postoperative quality of life in the three-port group was similar or even slightly higher than that in the five-port group. However, the difference was not significant (*P* > 0.05). Moreover, we found that the quality of life of the patients declined sharply after the operation, reached the lowest level at about 1 month after the operation, steadily recovered, and finally recovered to a slightly lower level than that before the operation in half a year.

## Discussion

Bladder cancer is a common malignant tumor of the urinary system, and RC remains the standard treatment [5]. With recent developments in science and technology, RC has gradually become minimally invasive, and most approaches have changed from traditional open surgery to the present LRC and RARC [5, 17]. Considering economic and other factors, LRC is still the preferred choice in most developing countries and in patients who are unwilling to bear high costs [9, 10]. In line with the concept of minimally invasive and concise surgery, Professor Bi of our research group improved and optimized LRC based on the traditional five-port method and adopted the three-port method [14]. Therefore, the clinical data of 100 patients undergoing LRC were collected in this study. The clinical effects of three-port LRC and traditional five-port LRC were compared by statistical analysis. Our study preliminarily showed that the three-port method was safe and feasible for patients who underwent traditional five-port LRC + ONB. We did not observe significant changes in perioperative data and postoperative complications, quality of life, or tumor outcomes; however, the cost of treatment was significantly reduced in patients who underwent the three-port procedure.

With continuous improvements in medical levels, minimally invasive and concise operations have received increasing attention. In this study, under the premise that the perioperative and postoperative clinical effect of three-port LRC is almost no worse than that of the five-port procedure, the number of cannulas and surgical cost can be reduced, which is quite valuable. In our research group's previous study on the learning curve, it was found that the sample size of the three-port procedure performed in our hospital steadily increased over time. As the number of cases increased and physicians became more skilled, the operative time decreased significantly [15]. This is consistent with the increase in the proportion of the three-port approach over the years in this study. The retrospective study of renal cancer conducted by Cheung et al. [18] also demonstrated the importance of minimally invasive trends in urology. They found that the proportion of minimally invasive procedures increased significantly over the years. In addition, as time has gone on and the volume of operations has increased, the techniques of doctors using minimally invasive procedures have also matured. We have been trying to accomplish the same goal with minimally invasive surgery in cases of bladder cancer. In the final analysis, these innovative minimally invasive attempts all follow the law of innovation diffusion, which is a principle describing the process and speed of new technologies and new attempts at their spread into society that has been verified in many disciplines [19–21]. According to this law, both the volume of surgery and the year of surgery are significant variables in the innovation and early trial phases, which are characterized by key discipline leaders proposing and leading the development of new technologies.

Because some patients were reluctant to undergo the innovative three-port procedure and others were reluctant to undergo more abdominal incisions, our team operated with different numbers of cannulas in different patients. The three-port method we used only required a primary surgeon and a laparoscopic assistant, while traditional LRC mostly employed the five-port method, which was jointly performed by three doctors. Although the traditional surgical method is quite classic, it still has some shortcomings, such as poor cooperation between different doctors, complicated operations, and high cost [5, 6, 22]. Three-port LRC is more in line with the concept of minimally invasive surgery and aesthetic needs. Fewer surgeon demands would allow for better allocation of fewer available physician resources. A lower number of cannulas can also directly reduce patient health care costs, which was preliminarily demonstrated in this study. In principle, a smaller number of incisions may also lead to fewer incision-related complications and a shorter recovery time; however, these were not significantly different between the two methods, which may be related to many factors such as insufficient sample size. Nevertheless, many perioperative and postoperative clinical data of patients undergoing LRC using the three-port method in this study are equal or even slightly better than those of patients with the traditional five-port method, which is a positive and optimistic signal. We believe that expanding the research scale and improving the surgical techniques will further reveal the advantages of the three-port method, being in line with the trend of simplified surgery and minimally invasive concepts. In summary, our study preliminarily suggests that the three-hole method may have the following advantages: (1) The perioperative and postoperative clinical data of the three-hole method are not significantly different from those of the traditional method, but the medical cost of the three-hole method is significantly reduced. (2) The three-hole method reduces the number of cannulas and is more concise and minimally invasive. It is thus in line with the aesthetic needs of patients and the trend of minimally invasive surgery. (3) The three-hole method reduces the need for surgeons and makes insufficient medical resources more reasonably distributed. (4) In addition, it is believed that with the increase in sample size and the progress of surgical technology, the advantages of the three-hold method, such as fewer trocars and less trauma, will become more statistically significant.

Of course, the three-port LRC also has many shortcomings. First, this surgical method has not been widely used worldwide, and there is no set of standardized procedures; therefore, it is quite a test of the skill and operation for the surgeon. Moreover, it is more difficult and time-consuming to learn. Our previous study on the learning curve also shows that LRC with the three-port method requires familiarity with a large sample size to be completed well [15]. Second, due to the lack of assistance from another assistant, it is difficult to carry out three-port LRC for obese patients and other situations that are difficult to fully expose. For primary surgeons with limited surgical experience and understanding, three-port LRC may even be less safe and reliable than the traditional method. Therefore, we recommend this innovative surgical approach for surgeons with extensive surgical experience.

Innovations such as three-port LRC to reduce the number of cannulas have been used in other urological procedures. Xu et al. [23–25] performed several cases of three-port laparoscopic radical prostatectomy (LRP). Their study successfully demonstrated that three-port LRP has significant advantages in terms of perioperative data such as operation time and intraoperative blood loss compared with traditional surgery, which is worthy of popularization and application. Because of the sufficient sample size and longer investigation time, their study is more convincing; however, the advantages of the three-port method are consistent with ours. We also reviewed and referred to other published literature on LRC, as shown in Table 6. Together with other LRC literature, we found that the three-port procedure may have certain advantages in shortening the operation time and reducing the amount of intraoperative blood loss. With a reduction in the number of cannulas, the surgical trauma of patients will be reduced in theory. However, considering the technical limitations of surgeons, when the number of cannulas is lower than a certain number, it may increase the operation time, EBL, postoperative complications, and other factors. From a single port to five ports, we adopted the three-port procedure as a compromise.

**Table 6:** Overview of the world literature on LRC

**Reference**	**Number of** port**s (n)**	**Number of patients (n)**	**OT (min)**	**EBL (ml)**	**Hospital stay after operation (days)**	**Complications (%)**	**PSM (%)**
Horstmann et al. [26]	1	8	434	643	16	37.5	0
Ma et al. [27]	1	5	343.2	270	19.5	20.0	0
Angulo et al. [28]	2	20	335	337	9	30.0	5
Angulo et al. [29]	2	30	330	347.5	10	40.0	6.7
Huang et al. [16]	5	171	325	270	13.1	39.2	0
Zhang et al. [30]	5	152	283.6	428.6	15.5	51.6	6.6
Snow-Lisy et al. [6]	5	87	450	400	NA	39.0	6.6
Khan et al. [31]	5	58	316	480.7	16.1	27.0	4
Our series	3	100	255.7	101.7	11.7	53.0	0

The main methods of urinary diversion after RC include orthotopic neobladder (ONB), ileal conduit (IC), and cutaneous ureterostomy (CU). Choosing a permanent urinary diversion method to reconstruct the lower urinary tract that can not only protect the function of the upper urinary tract but also improve the quality of life after surgery is the primary challenge associated with RC [32]. These three surgical methods are related to each other and each has their own advantages and disadvantages. The choice between these has been the focus of debate among doctors, patients, and even the entire urology department [33]. Houtmann et al. pointed out that these three procedures are most commonly used for ONB, followed by IC [34]. In ONB, we use the intestinal tract to create a new bladder and implant it into the body, which not only improves the quality of life of patients after surgery but also meets their psychological and aesthetic needs [35]. In view of this, for patients with better physical fitness or higher postoperative quality of life requirements, ONB is likely to be a more acceptable way to divert urine flow. Therefore, all patients selected in this study underwent ONB. All of them underwent standardized pelvic lymph node dissection and extracorporeal construction of Studer ONB. Moreover, our group will also conduct further research on more operative methods of three-port LRC in the near future.

A significant reduction in treatment costs (*P* = 0.035) was the only statistically significant measure in this study. The lower number of cannulas and lack of statistically significant differences in other metrics contributed to this result, which was not surprising. Bladder cancer has been reported to have the highest lifetime treatment cost among all malignancies [36]. Additionally, we often see that patients have doubts and concerns about the cost of treatment in clinical practice. Sometimes, they change the treatment method or even give up surgery. Therefore, the cost of treatment is not a negligible factor in treating bladder cancer and other malignant tumors. Many studies have shown that RARC is costly but superior to ORC in terms of complications and postoperative recovery [6–10]. In this case, for hospitals that do not have universal access to RARC and those that cannot afford the high cost, LRC is likely to be the most effective treatment, which is relatively a compromise as well. In LRC, the change from the five-port to the single-port approach means a reduction in the number of cannulas; however, the lack of surgical space and the limitations of the surgeon's skills may lead to poor outcomes. In this respect, the three-port LRC adopted by our research group is still in the middle position. Whether the effect brought by this "compromise" is at the middle point or the highest point of the statistical curve of all treatment methods is believed to be further reflected with the development and popularization of this surgical method.

With the development of biopsychosocial medical models, health-related quality of life (HRQOL) of patients with cancer has become a hot research topic in the field of medicine. Previous studies on RC have mainly focused on surgical methods and complications, but the postoperative quality of life has rarely been discussed. In recent studies, HRQOL has become an indispensable indicator for research on RC [37, 38]. Our research group counted the related indicators of HRQOL within one year after surgery. Here, the quality of life of patients decreased to the lowest level around one month after the operation and rose steadily in the following six months. Moreover, we observed that compared with the conventional five-port method, patients with three-port LRC showed a flat or even slightly higher quality of life. Although this difference was not significant, this result is substantial considering that our study was limited by the sample size and survey time. This suggests that the three-port approach is a reasonable alternative to the conventional five-port LRC.

This study has some shortcomings and deficiencies. First, the three-port LRC method is not widely used at present, which requires the surgeon to have rich experience in surgery and a high understanding of the relevant anatomy and operation. In some cases where it is difficult to fully expose the pelvic space, the three-port method may be inferior to the traditional five-port method, where an assistant can be arranged to assist in exposure and separation. Second, the three-port LRC procedure with other urinary diversion methods should be studied further. In addition, our sample size was inadequate and selection bias was possible. Surgical techniques, medical instruments, and perioperative management must be continuously improved. Finally, we did not have enough time to investigate the feasibility and reproducibility of this type of laparoscopic surgery before it was widely accepted. We still need a longer time and larger sample size data, as well as experience reports from other surgeons, to further confirm its feasibility and reproducibility.

## Conclusions

In this retrospective study, we noted that perioperative data, postoperative complications, quality of life, and tumor outcomes were not significantly different between patients who underwent three-port LRC and those who underwent traditional five-port procedures. However, the treatment cost for patients who underwent the three-port method was significantly reduced. These data preliminarily suggest that the three-port method is likely to be equally safe and feasible for patients suitable for traditional five-port LRC + ONB. Moreover, we need a longer time and larger sample size for prospective studies to further prove the results of the study.

## Data Availability

We obtained relevant data and materials from the corresponding author in a reasonable way. Data regarding the study can be obtained by email from the corresponding author by biliangkuan118@yeah.net.

## Abbreviations

RC: Radical cystectomy
ORC: Open radical cystectomy
LRC: Laparoscopic radical cystectomy
RARC: Robot-assisted radical cystectomy
ONB: Orthotopic neobladder
OT: Operative time
EBL: Estimated blood loss
MIBC: Muscle-invasive bladder cancer
NMIBC: Non-muscle-invasive bladder cancer
BMI: Body mass index
ASA: American society of anesthesiology
TURBT: Transurethral resection of bladder tumor
PSM: Positive surgical margin
IC: Ileal conduit
CU: Cutaneous ureterostomy
FACT-Bl-Cys: Functional Assessment of Cancer Therapy-Bladder Cystectomy
BCI: Bladder Cancer Index
HRQOL: Health related quality of life
NA: Not available
